# Parent–youth conflict as a predictor of depression in adulthood: a 15-year follow-up of a community-based cohort

**DOI:** 10.1007/s00787-019-01368-8

**Published:** 2019-07-13

**Authors:** Iman Alaie, Sara Brolin Låftman, Ulf Jonsson, Hannes Bohman

**Affiliations:** 1 Department of Neuroscience, Child and Adolescent Psychiatry, Uppsala University, Uppsala, Sweden; 2 Department of Public Health Sciences, Centre for Health Equity Studies (CHESS), Stockholm University, Stockholm, Sweden; 3 Karolinska Institutet Center of Neurodevelopmental Disorders (KIND), Centre for Psychiatry Research, Department of Women’s and Children’s Health, Karolinska Institutet, and Stockholm Health Care Services, Stockholm County Council, Stockholm, Sweden; 4 Centre for Psychiatry Research, Department of Clinical Neuroscience, Karolinska Institutet, and Stockholm Health Care Services, Stockholm County Council, Stockholm, Sweden

**Keywords:** Adolescent depression, Family conflict, Adulthood, Longitudinal design, Cohort study, Epidemiology

## Abstract

Experiencing conflictual relations with one’s parents while growing up has been linked to onset, recurrence, and worse treatment outcome of adolescent depression. While this suggests that significant problems in the parent–youth relationship make depressive disorders more relentless, it is not clear whether this effect lasts into adulthood. Our aim was to examine if major and minor conflict with parents while growing up predicts depression in adulthood in youth with and without a history of depression. We utilized data from the Uppsala Longitudinal Adolescent Depression Study. This community-based cohort was assessed with structured diagnostic interviews both at age 16–17 and at follow-up 15 years later. The analyses included 382 individuals (227 with a history of child or adolescent depression; 155 peers without such a history). Binary logistic regression was used, adjusting for sex, disruptive behavior disorders, and additional family-related adversities. Among individuals with adolescent depression, major conflict with parents was strongly associated with adult depression (adjusted OR 2.28, 95% CI 1.07–4.87). While major conflict with parents was rare among non-depressed controls, a non-significant association of similar magnitude was still observed. Minor conflict, on the other hand, was not significantly associated with adult depression. Overall, conflict with parents did not predict adult anxiety disorders, substance use, suicidal behavior, somatoform disorders, or psychotic disorders. In conclusion, major parent–youth conflict during upbringing seems to be linked with an increased risk of depression in adulthood. These findings underscore the need to consider contextual/familial factors in the prevention and clinical management of early-life depression.

## Introduction

Depression is common worldwide and has been recognized as the leading cause of disability among youths aged 10–24 years [1]. Early-onset depression has also been found to predict poor mental health outcome [2] and impaired psychosocial functioning [3] in adulthood, further underscoring the need for effective treatment and prevention. Despite the high burden of early-onset depression and recent advances in public awareness of mental disorders among youths, research indicates that the vast majority of those affected do not receive appropriate mental health care [4]. In addition, a substantial proportion of depressed adolescents do not respond to available evidence-based pharmacological and psychological treatments [5], or present with recurrent depressive episodes [6]. Similarly discouraging, the effect of indicated prevention seems to wear off over time [7]. There is therefore the need to identify relevant factors contributing to the persistence and treatment failure of adolescent depression, in order to inform potential targets for clinical intervention.

The role of family dynamics and interactional patterns, including discord, disagreement, and conflict in specific family systems (e.g., interparental, parent–child), has received increasing attention as regards factors relevant to early-onset depression [8, 9]. On the one hand, conflict in the parent–youth relationship can be viewed as normative during adolescence, and it has been suggested that moderate conflict with parents is linked to better adjustment as compared to either frequent or no conflict [10]. On the other hand, a small proportion of adolescents experience severely conflictual relations with their parents, though such extreme difficulties usually arise prior to adolescence [10]. As such, it may be the severity of conflict that is at play in the emergence of early-onset depression. There are several studies corroborating the link between parent–youth conflict and adolescent depressive symptoms, as indicated by both cross-sectional [11, 12] and longitudinal [8, 13–17] data, and recent research is indicative of both unidirectional [8, 18] and bidirectional [9, 15, 19] associations. Importantly, conflict with parents may trigger depression but could also be a consequence of the depression as such [9]. While the extensive line of research on the link between parent–youth conflict and adolescent depressive symptoms has yielded overall consistent findings, the underlying mechanisms and causative pathways are far from clear.

More to the point, recent sub-analyses of the Treatment of SSRI-Resistant Depression in Adolescents (TORDIA) study have shown that high levels of parent-reported conflict predicted a lower likelihood of remission in a sub-group of treated individuals [20]. However, intake family conflict in general did not predict remission, suggesting that the severity of the conflict is decisive. The results from the TORDIA study also lend support to potentially bidirectional influences between parent–youth conflict and depression, as remission of depression in fact was shown to reduce conflict. Similarly, in a randomized controlled trial comparing three forms of psychotherapy for adolescents with major depressive disorder, it was found that severity of depression and parent–youth conflict predicted lack of recovery, chronicity, and recurrence of depression at 2-year follow-up [21]. Taken together, these studies seem to imply that severe forms of parent–youth conflict may indeed be detrimental to adolescent mental health, possibly in a vicious circle. This further highlights the importance of considering parent–youth relationships as potential mechanisms of action of treatment.

While parent–youth conflict may be regarded as a risk factor for adolescent depression, there is less epidemiological evidence about the role of conflictual relations with parents for the continuity of depression across the life-course. Most prior longitudinal studies in this area included follow-up into young adulthood, but more often confined to mid-to-late adolescence [9]. This is to say that re-assessments frequently have been conducted shortly after baseline. In this short time frame, youths who have experienced adverse family climate are likely to be at risk for continued emotional distress, particularly if they are living at home with their parents. In the long-term perspective, on the other hand, the gradual increase in autonomy after the transition into adulthood may reduce any stress induced by conflict with parents, and thereby the risk of depression. The opposite is also possible, given that patterns of intergenerational continuity of high-conflict family environment have been reported [22], further perpetuating the sequela of maladaptive family dynamics. As such, the risk of recurrence or even first onset of depression might therefore continue into adulthood in those who have been exposed to conflictual relations with parents. Furthermore, it also remains to be seen whether the severity of parent–youth conflict impacts on adult depression specifically, or if the effects cut across other forms of adult psychopathology as well. It is noteworthy that studies of the long-term outcomes of adolescent depression have pointed to a distinctive role of early family-related adversities in the prediction of adult depression [23, 24] and bipolar disorder [24], as opposed to other adult mental health outcomes. Moreover, recent research using longitudinal nationwide register-based data has demonstrated a graded relationship between the number of childhood adversities and the subsequent risk for depression in young adulthood [25]. When examining the long-term association between parent–youth conflict and future depression it is therefore essential to adjust for potential confounders, including sex [26], disruptive behavior disorders [27], socioeconomic disadvantage [28, 29], parental separation [24], and parental depression [30, 31].

The aim of the present study was to evaluate the predictive association between parent–youth conflict and subsequent risk for depression in adulthood, also when controlling for potential confounders. Based on previous research, we formulated the following hypotheses:

1. For adolescents with a history of depression, conflict with parents while growing up is associated with depression recurrence in adulthood.
2. For adolescents with no history of depression, conflict with parents while growing up is associated with an increased risk of onset of depression in adulthood.
3. The association is evident primarily for major conflict with parents, as opposed to minor conflict.
4. Parent–youth conflict predicts adult depression specifically, as opposed to other mental disorders in adulthood.

## Methods

### Procedure

This study was based on the Uppsala Longitudinal Adolescent Depression Study (ULADS) [32], initiated in Uppsala, Sweden, in 1991–1992. Data from two distinct waves of data collection were utilized; a baseline wave at age 16–17 and a follow-up wave at approximately age 31–32. Initially, all students in the first year of upper-secondary school (ages 16–17 years), as well as same-aged school dropouts, were invited to participate in a screening for depression. Out of 2465 adolescents, 2300 (93%) underwent screening. Two self-report questionnaires were used: the Beck Depression Inventory-Child (BDI-C) [33] and the Centre for Epidemiological Studies-Depression Scale for Children (CES-DC) [34]. Participants with positive screening (BDI-C ≥ 16 [35] or CES-DC ≥ 30 + BDI-C ≥ 11 [36] or a self-reported suicide attempt) were subsequently assessed using the revised adolescent version of the Diagnostic Interview for Children and Adolescents (DICA-R-A) [37] according to the Diagnostic and Statistical Manual of Mental Disorders (DSM)-III-R criteria [38]. An equal number of individuals with negative screening, who were matched for sex, age, and school class, underwent an identical diagnostic assessment. In all, 307 screening-positive adolescents and 302 screening-negative controls participated in the baseline assessment and consented to be contacted for future follow-up.

The follow-up wave took place in 2006–2009. The formerly depressed and non-depressed adolescents who had been diagnostically assessed at baseline and who had consented to participate in a follow-up study were contacted. The follow-up investigation included the Mini International Neuropsychiatric Interview Plus (M.I.N.I. Plus) [39], which is a structured diagnostic interview based on DSM-IV criteria [40]. The follow-up also included several social and health-related aspects, and detailed questions about family history of mental disorders. In all, 409 of the initial 609 participants underwent the follow-up (67% retention rate). Further details about the data collection waves of the ULADS are described elsewhere [32].

### Participants

This study was based on the participants who took part at baseline and at follow-up assessments. Following a previously used categorization [24], participants with no identified lifetime depressive disorder and with no elevated depressive symptoms in adolescence were classified as controls, and contrasted against those who in adolescence had an identified lifetime depressive disorder or elevated depressive symptoms. Adolescents fulfilling criteria for mania or hypomania (*n* = 27), as assessed with DICA-R-A, were excluded due to the different trajectory and etiology of bipolar disorder. Consequently, the analyses of the present study are based on 382 individuals, of whom 227 presented with adolescent depression (124 with persistent depressive disorder; 63 with episodic major depressive disorder; 40 with subthreshold depression) and 155 were non-depressed controls.

### Measures

#### Parent–youth conflict

The Children’s Life Events Inventory (CLEI) [41], administered at baseline, provided self-reported information on life events while growing up. All items of the CLEI comprised dichotomous questions (i.e., yes/no) and participants were asked to respond if a particular event had occurred during the past year and/or earlier in life. The construct of parent–youth conflict was derived from two separate items asking if the participants had experienced minor and major conflict(s) with parents, respectively. Based on occurrences at any time point, three strata were created: participants who reported to have had no conflict, those who reported to ever have had minor (but not major) conflict, and those who reported to ever have had major conflict with parents.

#### Family income considerably reduced

This variable from the CLEI was used as a proxy for socioeconomic disadvantage in childhood. Participants who reported that their family income had been considerably reduced at any time point were contrasted against those did not answer in the affirmative.

#### Parental separation

This variable was derived from two items of the CLEI as to whether one’s parents had moved apart and whether one’s parents had divorced (and whether this had occurred during the past year and/or earlier in life). Those who reported that their parents had moved apart or divorced were coded as having separated parents.

#### Comorbid disruptive behavior disorders in childhood/adolescence

Lifetime diagnoses of attention-deficit hyperactivity disorder (ADHD), conduct disorder (CD), and oppositional defiant disorder (ODD*)* were assessed at baseline using DICA-R-A.

#### Parental depression

Based on information reported by the participants at the 15-year follow-up, when interviewed about family history of specific psychiatric disorders, this variable was coded as no vs. at least one parent having suffered from depression at any time point.

#### Depressive disorder in adulthood

This was operationalized as recurrent depression (i.e., at least two episodes) or one episode with a duration of at least 6 months from age 19 to 31, as assessed retrospectively at the follow-up using the M.I.N.I. Plus. This outcome measure has been used in previous publications [24, 42].

#### Other mental and behavioral disorders in adulthood

Diagnostic assessments of most other axis I disorders, according to DSM-IV, were also completed at follow-up using the M.I.N.I. Plus. These included lifetime and/or current diagnoses of, for instance, anxiety disorders, somatoform disorders, psychotic disorders, and substance use disorders, but also suicidal thoughts and behaviors.

### Statistical analysis

Binary logistic regression analyses were performed. Odds ratios (OR) with 95% confidence intervals (CI) are reported. In the descriptive analyses of conflicts with parents in adolescence and depression in adulthood, linear-by-linear associations were calculated. Any statistically significant association between parent–youth conflict and adult depression was further explored in sub-analysis examining the influence of when the conflict occurred (i.e., in the past year, earlier in life, or both). IBM SPSS Statistics version 25 was used to perform all the analyses.

### Ethical approval

The study was approved by the local ethical vetting board of Uppsala University, Sweden. All persons gave their informed consent prior to inclusion in the present study.

## Results

Descriptive statistics of the data material are presented in Table 1. In the total sample, 37.7% of the participants reported no conflict with parents in childhood/adolescence, 44.0% reported minor conflict, and 18.3% reported major conflict with parents. There were significant differences between depressed adolescents and non-depressed controls. Among the depressed, 27.3% reported major parent–youth conflict, as compared to 5.2% among the non-depressed controls. Furthermore, ADHD and CD/ODD were more common among the depressed than among the non-depressed. Adolescents with depression also more often had experienced that their family income had been reduced, and that their parents had separated. Furthermore, they were more likely to have a parent who had suffered from depression. In the total sample, 37.7% of the study participants had suffered from depression in adulthood. This was also more common among those who had been depressed in adolescence (48.5%) than among the non-depressed controls (21.9%).

**Table 1:** Descriptives

	All (*n *= 382)		Depressed adolescents (*n *= 227)		Non-depressed controls (*n *= 155)	
	*n*	%	*n*	%	*n*	%
*Baseline*						
Conflict with parents						
No	144	37.7	72	31.7	72	46.5
Minor	168	44.0	93	41.0	75	48.4
Major	70	18.3	62	27.3	8	5.2
Sex						
Males	79	20.7	47	20.7	32	20.7
Females	303	79.3	180	79.3	123	79.3
ADHD	26	6.8	25	11.0	1	0.7
CD/ODD	62	16.2	54	23.8	8	5.2
Family income reduced	64	16.8	49	21.6	15	9.7
Parental separation	136	35.6	94	41.4	42	27.1
Parental depression^a^	124	32.7	84	37.5	40	25.8
*Follow*-*up*						
Depression in adulthood	144	37.7	110	48.5	34	21.9

*ADHD* attention-deficit hyperactivity disorder, *CD* conduct disorder, *ODD* oppositional defiant disorder

^a^All *n *= 379; depressed *n *= 224

Table 2 presents the association between parent–youth conflict and depression in adolescence. The bivariate analyses showed that minor conflict was not significantly associated with depression in adolescence (OR 1.24, 95% CI 0.79–1.94). Major conflict was, however, strongly and significantly associated with adolescent depression (OR 7.75, 95% CI 3.46–17.34). All covariates except sex (which was one of the variables used in the matching procedure) demonstrated significant associations with adolescent depression. In the adjusted analyses, the estimate of major parent–youth conflict was attenuated but still strong and statistically significant (adjusted OR 4.69, 95% CI 2.01–10.97).

**Table 2:** Depression in adolescence regressed on conflict with parents (results from binary logistic regression analyses, *n *= 382)

	Bivariate^a^		Adjusted^b,c^	
	OR	95% CI	OR	95% CI
Conflict with parents				
No (ref.)	1.00	–	1.00	–
Minor	1.24	0.79–1.94	1.14	0.71–1.82
Major	7.75***	3.46–17.34	4.69***	2.01–10.97
Sex				
Males (ref.)	1.00	–	1.00	–
Females	1.00	0.60–1.65	1.30	0.73–2.32
ADHD	19.06**	2.55–142.21	14.00*	1.79–109.51
CD/ODD	5.74***	2.64–12.44	2.90*	1.25–6.76
Family income reduced	2.57**	1.38–4.77	1.63	0.82–3.26
Parental separation	1.90**	1.22–2.96	1.43	0.87–2.32
Parental depression^c^	1.73*	1.10–2.71	1.25	0.76–2.06

*ADHD* attention-deficit hyperactivity disorder, *CD* conduct disorder, *ODD* oppositional defiant disorder, *OR* odds ratio

***p < 0.001; **p < 0.01; *p < 0.05

^a^Bivariate includes one independent variable at the time

^b^Model adjusts for all independent variables simultaneously

^c^*n *= 379

The bivariate association between parent–youth conflict and depression in adulthood is reported in Table 3. In the total sample, among those who reported no parent–youth conflict, 28.5% suffered from depression in adulthood. The corresponding share for those who reported minor conflict was 36.3%, and for those reporting major conflict the share was 60.0%. When stratifying the analysis by depressed and by non-depressed controls, the pattern was shown to be similar across the two groups, although the proportions with adult depression were substantially larger among individuals who formerly also had suffered from adolescent depression.

**Table 3:** Depression in adulthood, by conflict with parents while growing up

	All (*n* = 382)		Depressed adolescents (*n* = 227)		Non-depressed controls (*n* = 155)	
	*n*	%	*n*	%	*n*	%
Conflict with parents						
No	41	28.5	27	37.5	14	19.4
Minor	61	36.3	44	47.3	17	22.7
Major	42	60.0	39	62.9	3	37.5
Linear-by-linear		17.76***		8.47**		1.00

***p < 0.001; **p < 0.01

To assess whether the bivariate association between parent–youth conflict and adult depression was robust also when adjusting for important and putatively confounding factors, we performed binary logistic regression analyses of depression in adulthood regressed on parent–youth conflict, whilst simultaneously adjusting for sex, ADHD, CD/ODD, reduced family income, parental separation, parental depression, and adolescent depression. The results, reported in Table 4, showed that major conflict was associated with depression in adulthood even when adjusting for these aforementioned factors. In the total sample, the only two predictors that demonstrated significant associations with depression in adulthood were major parent–youth conflict (adjusted OR 2.26, 95% CI 1.16–4.39) and depression in adolescence (adjusted OR 2.70, 95% CI 1.65–4.42). To assess whether the association between conflict with parents and adult depression varied between adolescents with and without depression, we included the interaction between conflict with parents and adolescent depression. However, a likelihood ratio test showed that this was not statistically significant. Nevertheless, in order to scrutinize the association between conflict with parents and adult depression in both groups, we performed stratified analyses among individuals with and without adolescent depression. In the analysis of formerly depressed adolescents, major conflict with parents was significantly associated with depression in adulthood (adjusted OR 2.28, 95% CI 1.07–4.87). A similar but not statistically significant tendency was observed among the non-depressed controls (adjusted OR 2.47, 95% CI 0.49–12.48). It should, however, be noted that the number of non-depressed controls who reported major parent–youth conflict was small (*n* = 8). In addition, relationships between the variables of parent–youth conflict and all other aforementioned variables in the model were subjected to further tests of interaction, but no evidence thereof was found.

**Table 4:** Depression in adulthood regressed on conflict with parents. Results from binary logistic regression analyses

	All^a^ (*n* = 379)		Depressed adolescents^a^ (*n *= 224)		Non-depressed controls^a,b^ (*n *= 154)	
	OR	95% CI	OR	95% CI	OR	95% CI
Conflict with parents						
No (ref.)	1.00	–	1.00	–	1.00	–
Minor	1.35	0.82–2.23	1.45	0.76–2.76	1.19	0.53–2.66
Major	2.26*	1.16–4.39	2.28*	1.07–4.87	2.47	0.49–12.48
Sex						
Males (ref.)	1.00	–	1.00	–	1.00	–
Females	1.32	0.74–2.35	1.23	0.61–2.50	1.58	0.54–4.62
ADHD	0.74	0.30–1.81	0.72	0.29–1.80	–	–
CD/ODD	1.68	0.88–3.20	1.59	0.77–3.24	1.99	0.41–9.55
Family income reduced	1.02	0.56–1.86	1.18	0.59–2.35	0.52	0.10–2.64
Parental separation	1.19	0.75–1.91	1.35	0.77–2.38	0.89	0.35–2.22
Parental depression	1.29	0.80–2.07	1.31	0.73–2.35	1.13	0.46–2.76
Depression in adolescence	2.70***	1.65–4.42	–	–	–	–

*ADHD* attention-deficit hyperactivity disorder, *CD* conduct disorder, *ODD* oppositional defiant disorder, *OR* odds ratio

***p < 0.001; **p < 0.01; *p < 0.05

^a^Model adjusts for all independent variables simultaneously

^b^ADHD omitted due to empty cells

Exploratory sub-analyses of the timing of conflict (i.e., in the past year, earlier in life, or both) were conducted in the full sample. The results revealed that participants who had reported major conflict with parents both in the past year and earlier in life (*n* = 14) were more likely to have depression in adulthood than those who reported having had no major conflict with parents (adjusted OR 4.54, 95% CI 1.18–17.44) (not presented in table). Those who had reported major conflict only in the past year (*n* = 29), or only earlier in life (*n* = 27), did not differ significantly from those with no major conflict.

Finally, we performed analyses of the full sample with parent–youth conflict as the predictor and a range of other mental health outcomes in adulthood based on the M.I.N.I. Plus (not presented in table). These analyses, adjusting for adolescent depression, did not show any significant associations of parent–youth conflict with subsequent anxiety disorders, somatoform disorders, psychotic disorders, suicidal behavior, or substance use. However, there was a statistically significant association between major conflict with parents and bipolar disorder in adulthood, even when adjusting for adolescent depression (OR 6.38, 95% CI 1.70–24.00) (not presented in table).

## Discussion

The goal of this longitudinal study was to examine if conflict with parents while growing up predicts subsequent depression in adulthood. While adolescent depression has been linked to recurrence later in life, this study demonstrated that depressed adolescents who reported major conflict with parents clearly were at an additionally increased risk of depression recurrence in adulthood. Even though major parent–youth conflict was infrequently reported by the non-depressed controls, a similar tendency was observed in this group. Of note, the severity of conflict seems to be crucial for the long-term mental health outcome as major, but not minor, parent–youth conflict was linked to subsequent depression in adulthood. As the highest risk of adult depression was observed in those who had reported experiences of major conflict in the past year and earlier in life, these results seem to suggest that both severity and duration of conflict are decisive for the long-term mental health outcome. Furthermore, it was found that major parent–youth conflict seems to predict both depressive and bipolar disorders in adulthood, as opposed to non-mood mental disorders. Importantly, the overall pattern of results was not accounted for by comorbidities commonly associated with interpersonal difficulties (i.e., disruptive behavior disorders), or other potential confounders related to contextual/familial adversities, such as parental depression.

The key finding that major parent–youth conflict was independently associated with adult depression may be interpreted in several ways. Conflictual relations with parents are indeed very stressful for many youngsters, not least since they depend on them as caregivers. Early negative parenting, through its proposed mechanism as a chronic stressor, may increase vulnerability to depression by lowering the threshold for affective problems later in life, potentially due to stress sensitization [30]. Research indicates that adverse parent–youth relations predict a long-lasting negative impact on later health, such that exposure to harsh parenting increases the risk for elevated depressive symptoms and inflammation in adulthood [43]. There is still, however, the need to clarify the role of early stress and its effects on health in the long run.

A related potential mechanism is the well-documented construct of expressed emotion (EE), which in recent theorizing and empirical work has been conceptualized as a form of toxic family stress [44]. EE is defined by attitudes of high criticism, hostility, and/or emotional over-involvement in the caregivers of a person suffering from a mental illness, and these may potentially be features that contribute to and maintain severe parent–youth conflict. The emphasis in EE is on dyadic relationships, highlighting the aversive patterns of interaction between caregiver and offspring without ascribing causative roles. High parental EE has consistently been found to be a risk factor for several forms of youth psychopathology, and has also emerged as a robust predictor of long-term outcome across a wide range of adult psychiatric disorders. As reported in a recent review paper [44], the rate of high parental EE is up to 51% among youth with major depression and with high familial risk for depressive disorder. EE has also been associated with the persistence of mood symptoms over time in both major depression [45] and bipolar disorder [46]. Importantly, it has been suggested that EE may moderate the course of early-life mental illnesses. As our study found a link between major parent–youth conflict and adult depression and bipolar disorder in formerly depressed adolescents, there may be certain overlapping symptoms in the early-onset mood spectrum disorders (e.g., irritability) that are particularly relevant to the emotional exchange and interactional patterns between adolescents and their parents. This finding is thus in line with the available evidence [9, 44–46], as major conflict with parents can be viewed as a more palpable consequence of an overall high EE in families of depressed youth. Yet, it remains to be determined exactly when and how maladaptive family dynamics may interact with individual susceptibility to future mood disorder. Relatedly, there is still the need to clarify if and how buffering protective factors might remedy the long-term mental health outcome.

Behavioral repertoires shaped early in life may also have contributed to increased vulnerability for depression recurrence in adulthood. Children and adolescents who have experienced conflictual relations with their parents would reasonably, as a result of observational learning, be less likely to acquire effective strategies for resolving or deescalating conflicts [47]. Irrespective of the causative factors and pathways, however, early experiences of maladaptive family dynamics and conflictual relations seem to disrupt normal development and aggravate emotional adjustment to adult life.

Given that minor parent–youth conflict predicted neither adolescent nor adult depression, the results of this study altogether point to the relevance of assessing the severity and duration of family conflict. Potentially, prolonged exposure to stressful interpersonal contexts may be of particular importance for the long-term vulnerability to affective problems. Furthermore, although minor parent–youth conflict was relatively common in the data used for this study, more importantly, our findings also suggest that minor parent–youth conflict in itself does not seem to have any substantially detrimental effect on mental health. The subjective perception of the conflict severity seems, however, to be of high clinical relevance, inasmuch as adolescents are able to differentiate their past and current experiences of minor and major parent–youth conflicts, respectively.

### Strengths and limitations

Strengths of this study include first and foremost its longer-term perspective, as participants were followed up after the transition into adulthood. The inclusion of a comparatively large cohort of adolescents recruited in the community, presumably covering a majority of actual cases with adolescent depression, has the potential of increasing generalizability of findings. Furthermore, all participants were assessed using validated self-report questionnaires and structured diagnostic interviews both at baseline and at the 15-year follow-up, which arguably is important from the perspective of clinical implications. However, our study is not without limitations. First, an important shortcoming concerns the measurement of parent–youth conflict, as only adolescents’ self-reports were collected. It thus remains an open question to what extent the parents would have confirmed the adolescents’ views. Recent research has recognized that reports from multiple informants imply certain methodological advantages, as disagreeing viewpoints offer valid information with regard to how the parent–youth relationship is perceived. This has highlighted the issue of informant discrepancies [48]. Second, there is the risk that current mental health status may have impacted on the perception of conflict severity. This may hypothetically always be the case whenever certain self-evaluations are filled in by afflicted individuals. Third, while research supports a bidirectional relationship between family conflict and youth depression, our study did not allow to disentangle this issue further. Fourth, the influence of the duration of parent–youth conflict was examined using only rough approximations about temporal occurrences of conflict. Fifth, the study was not without attrition, as approximately two-thirds of the original sample took part in the 15-year follow-up assessment. However, the participation rates were about the same among the formerly depressed and non-depressed adolescents. Finally, our study was somewhat underpowered to detect relevant differences among the formerly non-depressed controls. Our estimates were more precise for those afflicted with adolescent depression, but a non-significant association of similar magnitude was also observed among the non-depressed controls. While this tentatively signals that major parent–youth conflict might be harmful regardless of exposure to early-life depression, replication in future prospective studies is needed.

## Conclusion

In conclusion, this longitudinal study of a community-based cohort suggests that early-life experiences of highly conflictual relationships with parents have more enduring mental health consequences than previously known. Given that recent treatment research has been suggestive of a link between major parent–youth conflict and poor treatment response among depressed adolescents [20, 21], targeted strategies addressing adverse family climate could be a useful complement to existing prevention and treatment interventions in specific cases. Future intervention research should clarify if, how, and when such a complement should be used to optimize current best practice.
